# Characterization of the vaginal microbiota of healthy Canadian women through the menstrual cycle

**DOI:** 10.1186/2049-2618-2-23

**Published:** 2014-07-04

**Authors:** Bonnie Chaban, Matthew G Links, Teenus Paramel Jayaprakash, Emily C Wagner, Danielle K Bourque, Zoe Lohn, Arianne YK Albert, Julie van Schalkwyk, Gregor Reid, Sean M Hemmingsen, Janet E Hill, Deborah M Money

**Affiliations:** 1 Department of Obstetrics and Gynecology, University of British Columbia, 1190 Hornby Street, Vancouver, BC V6Z 2K5, Canada; 2 Women’s Health Research Institute, 4500 Oak Street, Vancouver, BC V6H 3N1, Canada; 3 Department of Veterinary Microbiology, University of Saskatchewan, 52 Campus Drive, Saskatoon, SK S7N 5B4, Canada; 4 Agriculture and AgriFood Canada, 107 Science Place, Saskatoon, SK S7N 0X2, Canada; 5 Department of Microbiology and Immunology, University of Western Ontario and Lawson Health Research Institute, London, ON, Canada; 6 National Research Council Canada, 110 Gymnasium Place, Saskatoon, SK S7N 0W9, Canada; 7 Department of Microbiology & Immunology, University of Saskatchewan, Saskatoon, SK S7N 5E5, Canada

**Keywords:** Vaginal microbiome, *cpn*60, Menstrual cycle, Bifidobacteriales, *Bifidobacterium*, *Lactobacillus*, Mollicutes, *Mycoplasma*, *Ureaplasma*, *Gardnerella*, Vaginal bacteria

## Abstract

**Background:**

The vaginal microbial community plays a vital role in maintaining women’s health. Understanding the precise bacterial composition is challenging because of the diverse and difficult-to-culture nature of many bacterial constituents, necessitating culture-independent methodology. During a natural menstrual cycle, physiological changes could have an impact on bacterial growth, colonization, and community structure. The objective of this study was to assess the stability of the vaginal microbiome of healthy Canadian women throughout a menstrual cycle by using *cpn*60-based microbiota analysis. Vaginal swabs from 27 naturally cycling reproductive-age women were collected weekly through a single menstrual cycle. Polymerase chain reaction (PCR) was performed to amplify the universal target region of the *cpn*60 gene and generate amplicons representative of the microbial community. Amplicons were pyrosequenced, assembled into operational taxonomic units, and analyzed. Samples were also assayed for total 16S rRNA gene content and *Gardnerella vaginalis* by quantitative PCR and screened for the presence of Mollicutes by using family and genus-specific PCR.

**Results:**

Overall, the vaginal microbiome of most women remained relatively stable throughout the menstrual cycle, with little variation in diversity and only modest fluctuations in species richness. Microbiomes between women were more different than were those collected consecutively from individual women. Clustering of microbial profiles revealed the expected groupings dominated by *Lactobacillus crispatus, Lactobacillus iners,* and *Lactobacillus jensenii*. Interestingly, two additional clusters were dominated by either *Bifidobacterium breve* or a heterogeneous mixture of nonlactobacilli. Direct *G. vaginalis* quantification correlated strongly with its pyrosequencing-read abundance, and Mollicutes, including *Mycoplasma hominis, Ureaplasma parvum,* and *Ureaplasma urealyticum*, were detected in most samples.

**Conclusions:**

Our *cpn*60-based investigation of the vaginal microbiome demonstrated that in healthy women most vaginal microbiomes remained stable through their menstrual cycle. Of interest in these findings was the presence of Bifidobacteriales beyond just *Gardnerella* species. Bifidobacteriales are frequently underrepresented in 16S rRNA gene-based studies, and their detection by *cpn*60-based investigation suggests that their significance in the vaginal community may be underappreciated.

## Background

It has long been recognized that the microbial community of the lower genital tract plays a vital role in maintaining the reproductive health of women [1]. The vaginal microbiota of reproductive-aged women has traditionally been characterized by culture-based techniques as dominated by *Lactobacillus* species, which, among other roles, produce lactic acid, biosurfactants, hydrogen peroxide, and other factors that create an inhospitable environment for pathogenic bacteria [2-7]. Detailed community profiling with culture-independent techniques has demonstrated that “healthy” microbial communities are usually dominated by *Lactobacillus crispatus, Lactobacillus iners, Lactobacillus gasseri, Lactobacillus jensenii,* or a combination of these species, and in a small portion of women, “mixed” profiles are depleted of lactobacilli and consist of bacteria such as *Gardnerella, Prevotella, Atopobium, Megasphaera,* and *Streptococcus*[8-14]. The latter has challenged our traditional understanding of the “healthy vaginal microbiome,” and raises questions about the structure and function of this community and the host response to it.

The relative compositional stability of the vaginal microbiome is quite remarkable, given the variability in the host ecosystem associated with the menstrual cycle, sexual contact, and introduction of bacteria from the skin and external environment. In particular, the menstrual cycle creates an ever-changing vaginal environment, with ovulation, menses, and corresponding fluctuations of estrogen and progesterone levels affecting bacterial attachment to the vaginal epithelium [15], cervical mucus production [16], pH and redox potential [17], and glycogen levels [18]. Culture-based studies in which the microbial community is characterized at several time points over the cycle have reported a range of findings, from no change in community composition, to some variation in the aerobic versus anaerobic communities over a menstrual cycle, and a greater proportion of non-*Lactobacillus* species present during menses [19-23]. This interindividual variability is mirrored in culture-independent studies, with some women maintaining a consistent microbial community throughout multicycle sampling, others having fluctuations timed with menses, and some having random fluctuations with no apparent cause [13,24,25]. Interestingly, in at least some cases, the overall functional characteristics of the community are predicted to be maintained, despite the fluctuations in the bacterial composition, because the shifts in relative dominance may be limited to different lactic acid-producing bacterial species [13].

Our understanding of the vaginal microbiota to date had been predominantly shaped by culture-based or 16S rRNA gene culture-independent studies. Although both approaches contribute a wealth of information on the microbiota composition, they have limitations. The move to culture-independent studies was spurred by the labor-intensive methodologies associated with bacteriologic culture (impractical for large studies), and limited ability to grow the diverse array of organisms present, as well as substantial difficulty in accurate determination of relative abundance. 16S rRNA gene-based methods have overcome some of the limits of culture, but are known to have amplification biases and offer limited resolution for some taxa [26]. Alternative molecular targets, like the universal *cpn*60 gene [27], have been used to gain a different perspective of microbial communities in a culture-independent fashion.

Similar to 16S rRNA gene-based studies, *cpn*60 surveys of the vaginal microbiome reveal many largely lactobacilli-dominated profiles, and better taxonomic resolution of some nonlactobacilli groups like *Gardnerella* and *Prevotella*[28-31]. For future studies of the vaginal microbiome, it is very important to understand whether timing of sampling during the menstrual cycle would result in significant variability that must be accounted for in cross-sectional studies of the vaginal microbiome in populations.

The primary objective of this study was to characterize the vaginal microbiome over a natural menstrual cycle among a cohort of healthy, asymptomatic, Canadian women, by using the *cpn*60 gene target. Secondarily, we aimed to probe for Mollicutes and Bifidobacteria not well detected by other high-throughput sequencing methods, and to better understand the temporal stability and/or variation of the vaginal microbiome within individual women.

## Methods

### Participants and study design

This longitudinal study was designed to collect vaginal-swab samples from a cohort of healthy women weekly over a single menstrual cycle. Women were eligible to participate if they demonstrated comprehension of the English language to a level necessary to provide informed consent, were at least 18 years of age, and had regular menstrual cycles. Individuals were excluded if they were pregnant or planning to become pregnant during the study period, had a chronic autoimmune or inflammatory condition, had an intrauterine device *in situ*, used hormonal contraceptives, or were currently taking or had taken antimicrobial medications (for example, antibiotic or antifungal therapy) within 4 weeks of enrollment. This study received ethics approval from the Institutional Review Board at the University of British Columbia (certificate no. H09-00860). The sample size was based on previous longitudinal studies of the vaginal microbiome in which a range of seven to 49 subjects provided sufficient numbers for microbiome investigation [13,25,32-34].

### Data and sample collection

Healthy reproductive-aged women were recruited from two cohorts of participants in Phase-III clinical trials of HPV vaccines, a private Obstetrics and Gynecology practice, and through online and print advertisements placed in Vancouver, British Columbia, Canada. After obtaining informed consent, basic demographic and clinical data were collected by history and from clinical records. While conducting routine speculum examination (usually for pap smear screening), clinicians used a Dacron swab (Copan Diagnostics Inc., Murrieta, CA, USA) to sample the posterior fornix and lateral vaginal wall. This sample represented the first of the four samples collected through the menstrual cycle from charts. All participants were provided with diaries to log activities including sexual activity and any interval symptoms. They were also provided with self-collection kits that contained sterile flocked swabs designed with a break point on the handle (Puritan Medical Products Company LLC, Guilford, ME, USA), collection tubes containing 200 μl of DNAzol-Direct reagent (MRCGENE, Cincinnati, OH, USA) and detailed collection instructions. After hand washing, women were instructed to insert the swab into their vaginas to the half-way point on the handle, rotate the swab 3 times, remove the swab and place it into the provided collection tube, break off the handle, leaving the swab head in the reagent tube, and close and date the tube.

Samples were stored at ambient temperatures until all three self-collected samples were acquired at 1-week intervals. This self-sampling method was duplicative of previous study methods used by ourselves and colleagues in which we validated the high quality of self-sampling compared with clinician sampling [35,36].

At the end of the collection period, participants returned samples and met with study staff to review their diaries and complete a final interview, in return for a modest honorarium ($20 CAD). All samples were de-identified, and by using information from participants assigned to a menstrual phase by using a calendar-based method: menstrual, day 1 (onset of menstruation) to cessation of bleeding (day 4 to 7); follicular, cessation of bleeding to day 12; periovulatory, day 13 to day 16; luteal, day 17 to days 26 to 32 (commencement of bleeding). If two samples were collected within the luteal phase, they were numbered sequentially as luteal-I and luteal-II. DNAzol-containing nucleic acid was separated from the swab head by centrifugation in the laboratory and used directly as template for PCR reactions.

### Quantitative PCR (qPCR) and conventional PCR

Samples were evaluated for nucleic acid integrity by quantification of the human cytochrome C oxidase subunit 1 (*cox*1) gene and bacterial 16S rRNA gene (V3 region) with SYBR Green assays, as described previously [37]. *Gardnerella vaginalis* was quantified with primers [38] and SYBR Green assay conditions [39] determined previously. The presence of Mollicutes (*Mycoplasma* or *Ureaplasma*) was determined by targeting the 16S rRNA gene by using a conventional, semi-nested PCR [40], and *Ureaplasma* spp. were detected by specific PCR for the multiple-banded antigen gene [41].

### cpn60 Universal Target (UT) PCR and pyrosequencing

PCR was carried out by using a cocktail of *cpn*60 UT-specific primers consisting of a 1:3 molar ratio of primers H279/H280:H1612/H1613, as described previously [29,42,43]. Primer sets were modified at the 5' end with one of 24 unique decamer multiplexing identification (MID) sequences, as per the manufacturer’s recommendations (Roche, Brandford, CT, USA). In addition, the Mollicutes-specific 16S rRNA gene PCR product from 12 samples was pooled and pyrosequenced. Amplicons were pooled in equimolar concentrations to create libraries for sequencing on the GS FLX Titanium platform. Emulsion PCR and sequencing were performed at the National Research Council, Saskatoon, SK, Canada.

### Analysis of operational taxonomic units (OTUs)

Pyrosequencing data were processed by using the default on-rig procedures from 454/Roche. MID-partitioned sequences were processed with the microbial Profiling Using Metagenomic Assembly (mPUMA) pipeline (http://mpuma.sourceforge.net[44]) with default settings to generate operational taxonomic units (OTUs) with gsAssembler (Roche). OTUs were screened and filtered for chimeras with Chaban Chimera Checker (C3), followed by manual curation. OTU abundance was calculated based on mapping of sequence reads to OTU sequences by using Bowtie 2 in mPUMA. OTUs were identified by watered-Blast comparison [29] to the *cpn*60 reference database, cpnDB_nr (downloaded on March 21, 2013, from http://www.cpndb.ca, [45]), and OTUs having the same best database reference were grouped together into nearest neighbor “species,” whereas OTUs having less than 55% identity to any reference sequence were removed from the dataset as non-*cpn*60 sequence. Raw sequence data files were deposited to the NCBI Short Read Archive (BioProject PRJNA210319). Samples with fewer than 100 sequence reads after processing were removed, leaving a total of 76 samples from 27 women for analysis.

### Statistical methods

We examined the demographic and clinical parameters of the study cohort by using descriptive statistics.

#### Alpha and beta diversity comparisons

Quantitative Insights Into Microbial Ecology (QIIME) pipeline [46] was used to calculate Shannon diversity index, Chao1 estimated number of species, and jackknifed beta diversity from Bray-Curtis distance matrices. For these analyses, the data grouped to the nearest neighbor “species” level were used, and all diversity measures were bootstrapped 100 times at 1,000 reads per sample or their sample maximum when less than 1,000. Rarefaction plots of Chao1 were generated to ensure that an adequate sampling depth for each sample was achieved.

Alpha diversity measures of the samples were averaged across the 100 bootstrapped datasets at 1,000 reads per sample, and compared among the four menstrual phases (menstrual, follicular, periovulatory, and luteal) by using a linear nested mixed-effects model for Shannon diversity and a Poisson nested mixed-effects model for Chao1 nested within subject. *Post hoc* Tukey comparisons were conducted when needed by using the “multcomp” package in R [47,48].

Beta diversity (ecologic distance) was calculated as the average pair-wise distances from the 100 bootstrapped datasets at 1,000 reads per sample. We compared ecologic distances of samples within subjects with those between by comparing all within-subject pair-wise distances with all between-subject pair-wise distances by using ANOVA. In addition, we compared all pair-wise distances within menstrual phases pooled (that is, all within menstrual, and all within follicular, all within periovulatory, and all within luteal) with all between-phase distances (again pooled) by using ANOVA. These analyses do not take into account multiple samples per subject, as they are based on pair-wise distances among samples; they should therefore be considered exploratory.

#### Hierarchic clustering

As the average of the bootstrapped distance matrices did not include the samples with <1,000 reads, we generated another Bray-Curtis distance matrix on the proportion of reads of each nearest neighbor “species” per sample by using only taxa that represented at least 1% of the reads of at least one sample by using the vegan package in R [49]. Hierarchic clustering was achieved by using this distance matrix and ward linkage. Bootstrap support for clusters was assessed by using average Jaccard similarities from the clusterboot function in the “fpc” package in R [50].

The results of the clustering are nearly identical when the bootstrapped averages for all samples with >1,000 reads are used instead. The additional samples contribute to some clusters, but do not change the structure of the clustering results.

#### Community stability

In addition to the linear mixed-effects models on alpha diversity, and the comparisons of within versus between-woman ecologic distances, we used a graphic method to assess the “stability” of microbial communities across samples from the same subject. To explore how the relative abundance of taxa changed over time, we plotted proportional area plots of taxonomic composition for each woman by using taxa that represented at least 10% of at least one sample. Women with samples that had <25% change in the proportions of dominant taxa were considered stable. Stability by this measure, and stability as a measure of whether a woman remained in one of the previously defined clusters, or switched among clusters, were tested for associations with BMI (underweight, normal, overweight, obese), previous pregnancy, marital status (partnered versus single), alcohol use, vaginal sex, use of condoms, and use of unscented tampons during the study period by using Fisher Exact tests.

#### Correlation of sequencing-read abundance to qPCR for Gardnerella vaginalis

The proportion of total pyrosequencing reads obtained per sample corresponding to *G. vaginalis* (nearest neighbor “species”) were compared with the absolute amount of *G. vaginalis* DNA detected by qPCR in each sample by using a Spearman rank-order correlation test (SPSS Inc., Chicago, IL, USA).

## Results

### Study cohort

Demographic and clinical characteristics of the 27 study participants are summarized in Table 1. All women had no signs or symptoms of a vaginal infection at the time of enrollment. All reported having normal menstrual cycles of approximately 28 days (range, 26 to 31 days). Most women (81.5%) reported clinical diagnosis of a vaginal infection (for example, yeast vaginitis, bacterial vaginosis (BV)) or a sexually transmitted infection (STI) at some time in their life. Only two women (7.4%) reported a diagnosis of BV in their lifetime, and only one woman (3.7%) reported having a BV episode within the past year. None of the women reported use of douche products during the study period.

**Table 1:** **Demographic and clinical data for study participants (*N*** **= 27)**

**Demographics**	
Age	34.96 ± 4.17 (18-53)
BMI	23.47 ± 1.86 (16.8-36)
Ethnicity	
Asian	9 (33.3%)
Caucasian	15 (55.6%)
Other	3 (11.1%)
**Substance use**	
Current smoking	2 (7.4%)
Current alcohol use	20 (74.1%)
**Sexual history**	
Marital status	
Partnered	9 (33.3%)
Single	18 (66.7%)
Sexual partners in past year	1.04 ± 0.28 (0-3)
Sexual partners in past 2 months	0.78 ± 0.23 (0-2)
Vaginal intercourse during study period	17 (63.0%)
Condom use during study period	8 (29.6%)
Previous pregnancy	10 (37.0%)
Surgical sterilization	4 (14.8%)
**Menstrual cycle**	
Cycle duration (days)	28.05 ± 0.68 (26-31)
Tampon use during study period	12 (44.4%)
**BV and STI history**	
Diagnosed with BV, yeast, or STI in lifetime	22 (81.5%)
Diagnosed with BV in lifetime	2 (7.4%)
BV episode in past year	1 (3.7%)
Antimicrobial use in past 2 months ^a^	
Oral	1 (3.7%)
Topical	2 (7.4%)

^a^No intravaginal antibiotics.

BMI, body mass index; BV, bacterial vaginosis;

STI, sexually transmitted infection.

Continuous variables are reported as means ± 95% CI (range).

Categoric variables are reported as N (%).

### Sample integrity evaluation

To confirm the presence of amplifiable nucleic acid in the DNAzol samples, samples were assayed for human cytochrome *C* oxidase gene and total bacterial 16S rRNA gene content. Human DNA was detectable in 72 (98.6%) of 73 samples tested, whereas total 16S rRNA gene content fell within the ranges of less than 10^4^ copies/swab (33 samples), 10^4^ to 10^5^ copies/swab (31 samples) or 10^5^ to 10^6^ copies/swab (12 samples). All samples generated *cpn*60-UT amplicons for pyrosequencing.

### Microbiome profile generation

After sample collection and processing, an average of three vaginal samples per woman (range, 1 to 4; median, 3) were available for analysis. Microbial profiles were determined from an average of 7,585 sequence reads per sample (range from 125 to 37,419; median, 4,256), generating 567 unique OTUs. When combined at the nearest neighbor “species” level (OTU with same best reference database match), 73 bacterial “species” were identified with an average minimum percentage identity of 94.0% ± 7.1%, an average maximum percentage identity of 96.6% ± 5.2%, and an average overall percentage identify of 95.5% ± 5.5% to the reference sequence. A detailed summary of the nearest neighbor “species” identified in each sample is provided in an additional file (see Additional file 1).

To evaluate whether the number of sequence reads per sample was a sufficient representation of the vaginal microbiota, rarefaction plots of Chao1-estimated number of species were calculated. Graphic depictions of the rarefaction plots for subsampled data are presented in an additional file (see Additional file 2). In most samples, the Chao1 values remained relatively consistent throughout (flat line across the plot), indicating that the sample richness did not change significantly when 100 to 1,000 sequence reads per sample were examined. This consistency indicated that most of the community was captured at the sampling depth achieved.

### Vaginal microbiome richness, diversity, and ecologic distance

The Shannon diversity index provides a quantitative measure of species diversity (richness and evenness), whereas the Chao1-estimated number of species provides a quantitative measure of species richness. No significant difference was found among the menstrual phases (linear mixed-effect model; likelihood ratio test, *P* = 1) in the average Shannon diversity of vaginal microbiome samples after accounting for multiple samples from each individual. However, a significant relationship between menstrual phase and the Chao1 estimated number of species was observed (Poisson mixed-effects model: likelihood ratio test, *P* = 0.03), with *post hoc* comparisons suggesting that the follicular phase had, on average, 1.3 more species than the luteal phase (adjusted *P* = 0.01), after multiple samples per individual were taken into account. None of the other menstrual-phase comparisons showed significant differences. Figures showing the average Shannon diversity index and Chao1 estimated number of species calculated for each menstrual phase are presented in an additional file (see Additional file 3).

**Figure 1:**
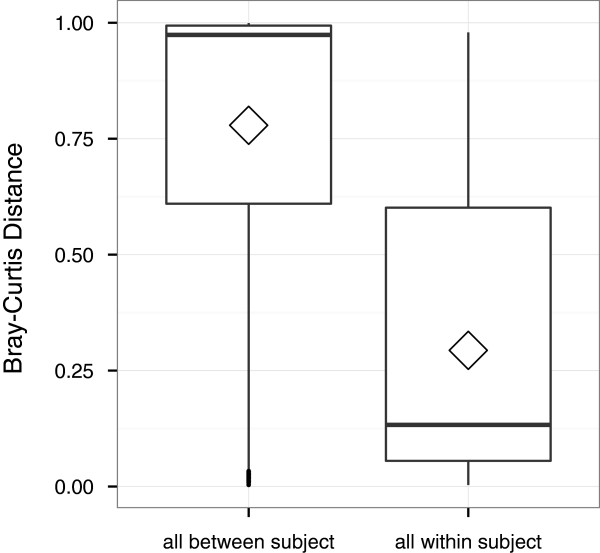
**Average Bray-Curtis ecologic distance from jackknifed distance matrices of vaginal microbiomes.** The horizontal lines indicate the median distances; boxes indicate the interquartile ranges; whiskers extend to 1.5 times the interquartile range; and the diamonds indicate means.

Comparisons of communities between vaginal microbiome profiles showed on average greater ecologic distance between samples from different women (median = 0.97; range = 0 to 1) than among samples from the same woman (median = 0.29; range = 0 to 0.98; Figure 1: ANOVA, *F*_1, 2,209_ = 133.4; P < 0.0001). No discernible difference in ecologic distance was noted when comparing samples between menstrual phases versus within menstrual phases.

### Vaginal microbiome composition

**Figure 2:**
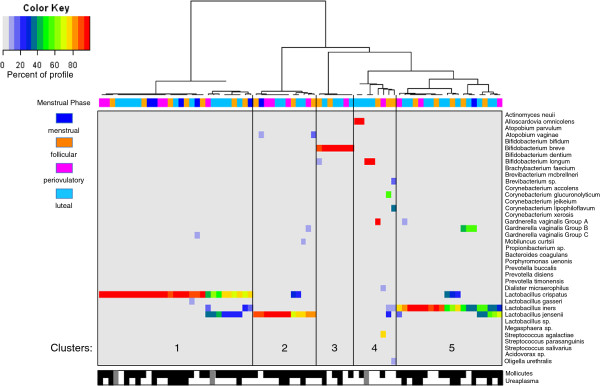
**Hierarchic clustering of vaginal microbiome profiles by nearest-neighbor “species.”** Only nearest-neighbor “species” comprising at least 1% of at least one sample are included. The color scale reflects the proportion of the total profile each “species” represented. Samples were divided into five clusters (indicated by numbers) based on average Jaccard similarities from 100 resampling runs. Colored blocks on the top indicate the menstrual phase of each sample. Mollicutes- and *Ureaplasma*-positive samples are indicated by black boxes below the heatmap (gray boxes indicate that the sample was not tested).

Hierarchic clustering of all 76 samples revealed five clusters with average Jaccard similarities from 100 resampling runs of 0.99, 0.85, 0.96, 0.80, 0.78 (left to right in Figure 2). Clusters 1, 2, 3, and 5 from left to right in Figure 2) were dominated by *L. crispatus* (29 samples from 13 women), *L. jensenii* (12 samples from five women), *Bifidobacterium breve* (a non-*Gardnerella* Bifidobacteriales: seven samples from two women), and *L. iners* (20 samples from 11 women). The remaining cluster contains a heterogeneous mixture of dominant taxa including *Alloscardovia omnicolens, Bifidobacterium longum, Streptococcus agalactiae*, *G. vaginalis* subgroup A, and mixed *Actinobacteria* species (eight samples from five women).

**Figure 3:**
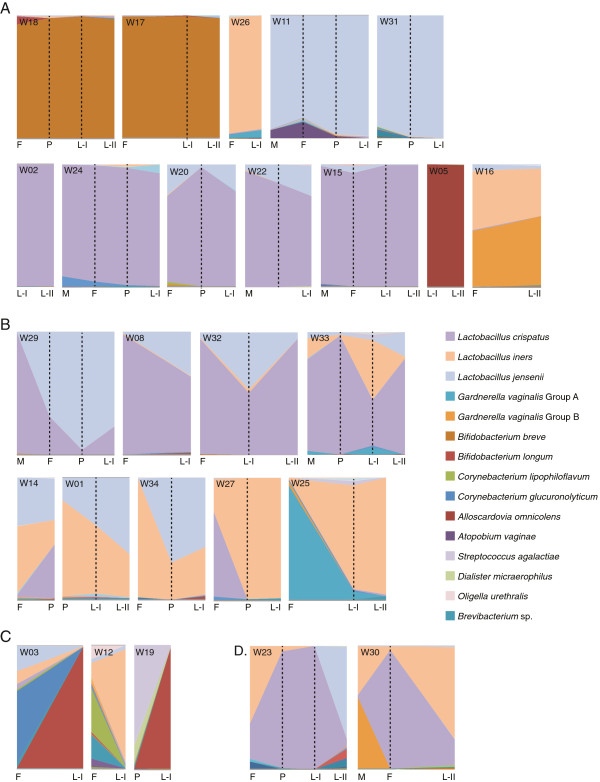
**Vaginal microbiome profiles over the menstrual phase.** Data are presented as proportion of the total sequence reads obtained for each sample, with the height of the ordinate corresponding to 100%. Sampling times are indicated with vertical broken lines, and menstrual-cycle phase for each sample is indicated on the abscissa (M, menstrual; F, follicular; P, periovulatory, L-I, luteal I; L-II, luteal II, as defined in the text). Profiles are arranged to reflect women with relatively stable microbiomes (<25% change) **(A)**, profiles consisting of the same organisms, but the proportions of these organisms fluctuated by >25% over time **(B)**, profiles that had dramatic changes over the sampling time **(C)**, and profiles with a mixture of changing proportions and introduction of new organisms **(D)**. Sample identification numbers appear in the upper left corner for each individual. The legend includes nearest-neighbor "species" that account for at least 10% of the sequence reads in at least one sample.

Of the 26 women who had multiple samples in the analysis, 18 had all their vaginal profiles belonging to the same cluster, whereas eight had different cluster affiliations over the menstrual cycle. Four of the women who had cluster switches (W14, W27, W30, and W33) transitioned between the *L. iners* cluster and the *L. crispatus* cluster and so remained *Lactobacillus* dominated overall. One woman (W29) transitioned between the *L. crispatus* and the *L. jensenii* cluster, and one woman (W23) transitioned between all three *Lactobacillus*-dominated clusters*.* None of the women transitioned into or out of the *B. breve*-dominated cluster, whereas two women (W12 and W25) transitioned from the heterogeneous cluster to a *Lactobacillus*-dominated cluster over the menstrual cycle (Figure 3).

A relation did not appear to be present between clustering and menstrual phase, as most clusters contained samples from all phases (Figure 2). An inventory of the dominant species profile identified from the different menstrual-phase samples provided by each individual is provided in a supplementary file (see Additional file 4).To gain a clearer picture of the stability of vaginal microbiome community composition over time, proportional area plots of the taxonomic composition of each woman’s microbiome were assembled (Figure 3). The plots grouped into four general types of vaginal microbiome profiles. The first group consisted of 12 women with relatively stable bacterial profiles over the sampling course (<25% variability in relative proportion of dominant bacterial species; Figure 3A). These women were also among those who did not switch between community clusters, as defined earlier by hierarchic clustering.The second group comprised nine women with microbiota profiles that consisted of the same bacterial organisms, but the relative proportions of each “species” fluctuated by >25% over time (Figure 3B). Five of these subjects also switched between clusters, as defined earlier, whereas the remaining four did not.The third group consisted of three women who showed dramatic changes in their microbiota over the sampling course (a complete change in the dominant members of the community; Figure 3C), whereas the fourth group contained two women with >25% variability in relative proportions of bacterial “species,” as well as an apparent introduction of new bacterial “species” (Figure 3D).

Differences in microbiome composition were reflected in analogous differences in the Shannon diversity index and Chao1-estimate values. Plots depicting temporal variation in the bootstrapped Shannon diversity index and Chao1 estimates for each individual are included in an additional file (see Additional file 5).

No evidence was seen that “stability,” as defined either as <25% change in dominant taxa, or as cluster-switching, was related to BMI category, previous pregnancy, marital status, alcohol use, vaginal sex, use of condoms, or use of unscented tampons during the study period by Fisher Exact tests (all *P* > 0.05). Other clinical covariates were not investigated, as too few subjects existed per category (Table 1).

### Evaluation of nearest-neighbor “species” abundance by G. vaginalis levels

In an effort to confirm independently whether the nearest-neighbor “species” sequence read abundance reflected actual bacterial species levels in the samples, *G. vaginalis* was quantified in the samples, and the quantitative PCR results compared with sequence read counts. *G. vaginalis* was chosen as a target because it was detected in 74% of samples at a wide range of sequence read abundances. The proportion of total reads obtained per sample corresponding to *G. vaginalis* was significantly correlated with the amount of *G. vaginalis* detected by qPCR in each sample (*r*_s_ = 0.405; *n* = 76; *P* < 0.001).

### Mycoplasma and Ureaplasma in the vaginal microbiome

A known limitation of *cpn*60-based microbial profiling is that some species of Mollicutes do not contain this gene. To address this issue, all samples were screened for Mollicutes by using a conventional PCR-based test designed to detect *Mycoplasma* and/or *Ureaplasma* species. Mollicutes were detected in 59 (80.8%) of 73 samples, representing 23 (85.2%) of 27 women (Figure 2). When a *Ureaplasma*-specific PCR was applied to the same samples, 23 (31.5%) of 73 returned positive results from 10 (37.0%) of 27 women, comprising of 18 samples (24.7%) with *U. parvum* (eight women) and five samples (6.8%) with *U. urealyticum* (two women). To understand the composition of the Mollicutes-level PCR products further, PCR products from 12 samples (representing five women in either follicular, periovulatory, or luteal phases) were pooled and pyrosequenced to generate 54,926 reads. Mollicutes-like sequences accounted for 77.7% of reads, with clear identities (>95%) to *Mycoplasma hominis* and *Ureaplasma* spp. observed for 76.2% of the sequences (*Ureaplasma* cannot be speciated with this region of the 16S rRNA gene). The remaining 1.5% of Mollicutes-like reads grouped into seven distinct OTU that had only 85% to 90% identity to known species, suggesting that novel Mollicutes may be present in the microbiome.

Sequences matching *Mycoplasma genitalium* were not detected. Interestingly, this PCR also generated amplicons from several *Staphylococcus* species, including *S. epidermidis* (17.3% of reads), *S. pasteuri* (0.21% of reads), *S. hyicus* (0.04% of reads), and *Staphylococcus* spp. (2.9% of reads), as well as the *Lactobacillus* species *L. iners* (1.6% of reads), *L. crispatus* (0.04% of reads), and *L. jensenii* (0.07% of reads). This indicated that some of the PCR product generated was not Mollicutes-derived and suggests that this PCR assay has the potential to generate a false-positive result.

## Discussion

The results of our *cpn*60-based study of the vaginal microbiota through a menstrual cycle demonstrate that in this cohort, only a few community states exist in healthy women, with *L. crispatus, L. iners,* and *L. jensenii* being the dominant members. This is consistent with previously reported studies [12,13,25]. Of note, *Lactobacillus gasseri* is prominent in other studies [12,13], but it was not a dominant organism in any woman’s microbiota profile in this study.

In overall richness, diversity, and ecologic distance, we observed an element of a personalized compositional pattern in most women over time: profiles generated from samples collected at different menstrual phases from the same woman showed a higher degree of shared similarity in community structure versus profiles generated from different women but collected in the same menstrual phase [13,51]. However, given the limited sample size, the extent of a “personalized” microbiota remains to be confirmed.

We did observe a statistically significant relation between menstrual phase and the Chao1 estimated number of species, in which follicular-phase samples had on average 1.3 more species than did luteal-phase samples. However, we do not believe that this observation is biologically significant, given that presence or absence of a single species can be affected by technical nuances and random sampling effects [52].

Genital *Mycoplasma* and *Ureaplasma* have been associated with female genital infections such as vaginitis, cervicitis, and pelvic inflammatory disease, and with a variety of other negative reproductive and neonatal outcomes [53-55]. *M. hominis, M. genitalium, U. parvum,* and *U. urealyticum* are regularly detected in vaginal samples by using culture or taxon-specific PCR methods, although the reported prevalence is wide-ranging (20% to 80% depending on the study population). These species are much more rarely reported in 16S rRNA gene-based microbiota analysis [12,14,51], likely because of universal primer bias [56]. The lack of a *cpn*60 gene in Mollicutes is not a universal phenomenon, because several species of *Mycoplasma,* including *M. genitalium* do have a *cpn*60 gene. However, *M. genitalium* has not been detected in any *cpn*60-based studies of the human vaginal microbiome to date [28-30]. *Mycoplasma* and *Ureaplasma* are commonly reported vaginal constituents [57,58], further supporting the suggestion that these organisms are part of the vaginal microbiota of many clinically healthy women. As well, deep sequencing revealed seven novel Mollicutes-like sequences that could indicate uncharacterized species. Interestingly, deep pyrosequencing of the reportedly Mollicutes-specific PCR products from a pool of 12 PCR-positive samples showed that primer specificity was imperfect. The generation of amplicon from other organisms, particularly *Staphylococcus* and *Lactobacillus* species, that was the same size as the targeted Mollicutes product might result in some overreporting of Mollicutes detection in studies using these primers.

Our study diverges from several others in the number of vaginal profiles dominated by non-*Gardnerella* Bifidobacteriales. *Bifidobacterium* species (high G + C, Gram positive, Actinobacteria) are known members of the vaginal microbiota, and genera like *Bifidobacterium* and *Alloscardovia* have been isolated from vaginal samples in several culture-based investigations [19,21,59-62]. Culture-independent studies based on the 16S rRNA gene have also reported *Bifidobacterium* species in the vaginal microbiome [14,63,64], with one study finding it the dominant community member for two of 20 women in the study [65]. More commonly, 16S rRNA gene-based investigations tend not to report *Bifidobacterium* in the vaginal microbiome [12-14,51]. This phenomenon echoes previous findings in studies of the intestinal microbiome, in which culture-based investigations revealed a wealth of *Bifidobacterium,* whereas 16S rRNA gene-based culture-independent studies detected very few, leading to the discovery that many universal 16S rRNA gene PCR primers were a poor match for this genus [26,66,67].

Further hindering the identification of *Bifidobacterium* is its relatedness to *G. vaginalis*, a species that is prevalent in the vaginal microbiota, and belongs to the same taxonomic family [68]. The *cpn*60-based universal PCR protocol used in this study has been shown experimentally to represent bifidobacteria more accurately in intestinal microbiomes compared with 16S rRNA gene-based universal PCR [26], and the *cpn*60 target sequence is clearly distinguishable between *Gardnerella* and *Bifidobacterium* (average sequence identity of only 75%, compared with 16S rRNA gene identities of >90%). The finding that five women in our study group had *Bifidobacterium breve, Bifidobacterium longum,* or *Alloscardovia omnicolens* as a dominant vaginal organism seems reasonable. Bifidobacteria are generally considered to be beneficial members of the intestinal microbiota [69], although their role in the vaginal microbiota has not yet been elucidated. It is conceivable that *Bifidobacterium,* lactic acid-producing bacteria, could have a protective or health-promoting effect in the vagina analogous to *Lactobacillus*. Bifidobacteria also appear to play an important role in early infant health and development [70], and their presence in the vaginal microbiota of healthy, reproductive-aged women could provide a means of transfer from the mother to the newborn during birth.

Measures were taken to evaluate sample quality, representativeness of the sequence read abundance, and known limitations of the technique used. Sample quality was assessed from the ability to amplify both human (*via* the *cox*1 gene) and bacterial DNA (via the 16S rRNA and *cpn*60 genes) from the samples. The fact that bacterial amplicon could be generated from all samples and human amplicon from all but one sample indicated that nucleic acid quality was maintained at a sufficient level for analysis. It was then determined empirically that *cpn*60 amplicon generation and sequencing faithfully represented actual starting amounts of target in the example case of *G. vaginalis*. In addition, it was anticipated that Mollicutes species would be poorly characterized by *cpn*60 analysis, so targeted investigation of this important group was separately undertaken. All of these measures added additional experimental data to the results generated from the *cpn*60 analysis and helped to ensure that the descriptions of the vaginal microbiota under investigation were as faithful and representative as possible.

The results of this study suggest that the specific menstrual phase of a woman is not predictive of her vaginal microbiota at that time. For 18 (69%) women, all the samples collected throughout the menstrual cycle clustered together by composition. An additional six (23%) women transitioned between *Lactobacillus-*dominated clusters throughout the menstrual cycle, whereas only two (8%) women had sample compositions transition from the heterogeneous cluster to a *Lactobacillus*-dominated cluster. No apparent pattern of vaginal microbiome composition change was noted by menstrual phase, and no association with personal health practices or sexual activity. It is worth noting that the majority of the women in this study had microbiota dominated by *Lactobacillus* species. Although this is a commonly observed trend, we recognize that the observations made from this cohort may not apply to populations of women with a higher prevalence of non-*Lactobacillus*-dominated vaginal microbiota. Further studies involving more Canadian women over longer periods are needed to continue addressing important questions about the composition and stability of the vaginal microbiome. It is also important to ensure that these studies are done in different cohorts with variable ethnic and behavioral characteristics in multiple sites around the world.

## Conclusions

The composition of the vaginal microbiome did not appear to be directly linked to the menstrual phases of women in our study. Some woman had vaginal microbial communities that were apparently stable in composition and abundant throughout the menstrual cycle, whereas others underwent moderate or dramatic shifts over time. Despite these shifts, the overall community composition tended toward one of only a few compositional structure types dominated by *Lactobacillus* species at any single time. The use of the protein-coding *cpn*60 gene as a community barcode marker, unlike some other methods, revealed Bifidobacteriales-dominated vaginal profiles whose role in health warrants further investigation. The degree to which specific aberrations of a woman’s vaginal microbiome occur and lead to symptomatic disease requires urgent attention, given the prevalence of such illnesses and its adverse effects on quality of life and reproductive outcomes.

## Abbreviations

16S rRNA: 16-Svedberg ribosomal RNA; BV: bacterial vaginosis; *cox*1: cytochrome *C* oxidase subunit 1 gene; *cpn*60: chaperonin-60 gene; *G. vaginalis*: *Gardnerella vaginalis*; G + C: guanine + cytosine; HPV: human papillomavirus; *L. crispatus*: *Lactobacillus crispatus*; *L. iners*: *Lactobacillus iners*; *L. jensenii*: *Lactobacillus jensenii*; *M. genitalium*: *Mycoplasma genitalium*; *M. hominis*: *Mycoplasma hominis*; MID: multiplexing identification; mPUMA: Microbial Profiling Using Metagenomic Assembly; NCBI: National Center for Biotechnology Information; OTU: operational taxonomic unit; PCR: polymerase chain reaction; QIIME: Quantitative Insights Into Microbial Ecology; qPCR: quantitative polymerase chain reaction; *S. epidermidis*: *Staphylococcus epidermidis*; *S. hyicus*: *Staphylococcus hyicus*; *S. pasteuri*: *Staphylococcus pasteuri*; *U. parvum*: *Ureaplasma parvum*; *U. urealyticum*: *Ureaplasma urealyticum*; UT: universal target.

## Competing interests

The authors declare that they have no competing interests.

## Authors’ contributions

DM, JvS, GR, SMH, and JEH conceived the study, oversaw and contributed to data collection and analysis, and participated in manuscript writing. BC generated 16S rRNA and *Gardnerella* qPCR data, and participated in data analysis and manuscript writing. MGL did data analysis and participated in manuscript writing. TPJ generated the *cpn*60 vaginal microbiome profiles and Mollicutes data. ECW oversaw clinical data collection and analysis and participated in manuscript writing. ZL participated in manuscript writing. AYKA did statistical analysis and participated in manuscript writing. DB oversaw clinical sample and data collection. All authors read and approved the final manuscript.

## Supplementary Material

Additional file 1 **Detailed summary of nearest-neighbor species identified in each sample.** Summary table of nearest-neighbor “species” identified in each sample, minimum, maximum, and average percentage identity of the nearest-neighbor “species” label to database reference sequences, number of OTU (unique *cpn*60 sequence) per nearest neighbor “species,” and actual nearest-neighbor “species” sequence abundance (pyrosequencing reads obtained) for each sample. Click here for file

Additional file 2 **Rarefaction plots of Chao1-estimated numbers of species values for all 76 study samples.** For each sample, 100 to 1,000 sequence reads (in increments of 25 sequences) were subsampled 100 times from the data, and the average Chao1-estimate numbers of species were plotted for each increment. If a study sample had been thoroughly sequenced, the data plotted would approach an asymptotic plateau, indicating that further sequencing would not yield significantly more new species. This was done to confirm that the sequencing depth used in this study was adequate to capture the sample richness. Click here for file

Additional file 3 **Average Shannon Diversity and Chao1-estimated numbers of species by menstrual phase.** Average Shannon Diversity **(A)** and Chao1-estimated number of species **(B)** by menstrual phase. Phases are defined as menstrual, day 1 (onset of menstruation) to cessation of bleeding (days 4 to 7); follicular, cessation of bleeding to day 12; periovulatory, day 13 to day 16; luteal, day 17 to day 26 to 32 (commencement of bleeding). The error bars indicate 95% confidence intervals. The only statistically significant difference determined was between the Chao1-estimated numbers of species between the follicular and luteal phases; however, we do not believe this to be biologically significant. Click here for file

Additional file 4 **Cluster affiliation for all 76 study samples.** Summary table identifying the hierarchic clustering assignment of all 76 samples into one of five clusters based on Jaccard similarities. These data are depicted graphically in Figure 2. Click here for file

Additional file 5 **Temporal variation in Bootstrapped Shannon diversity index and Chao1 estimates for each individual.** Graphs showing the change in bootstrapped Shannon diversity index **(A)** and Chao1 estimates **(B)** for each woman (*N* = 27) by the day each sample was taken. The plots reflect the findings that many women had diversity statistics that remained consistent throughout the study period, whereas some women had changes in the values of these measures. Click here for file
