# Supplementary figures and images for: Characterization of the vaginal microbiota of healthy Canadian women through the menstrual cycle
Source: Microbiome. 2014 Jul 4;2:23. doi: 10.1186/2049-2618-2-23 (PMC4106219; doi:10.1186/2049-2618-2-23)

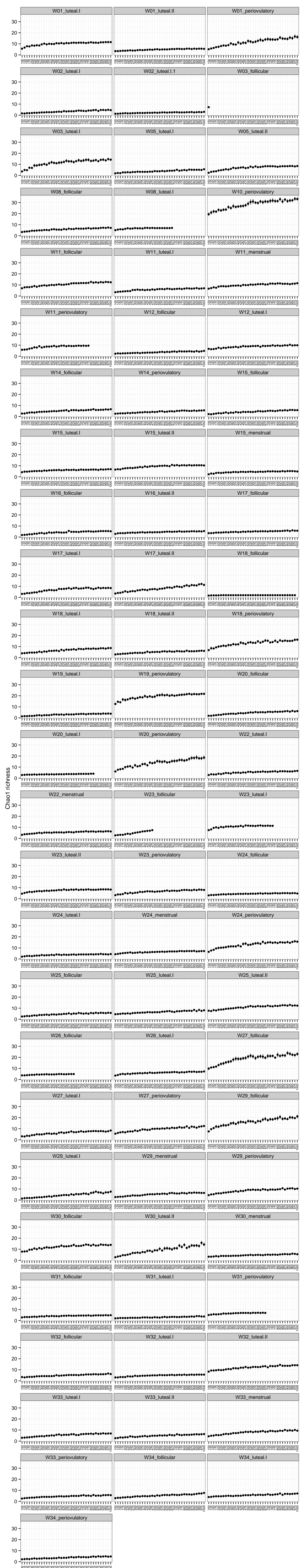

Supplement: Additional file 2 — Rarefaction plots of Chao1-estimated numbers of species values for all 76 study samples. For each sample, 100 to 1,000 sequence reads (in increments of 25 sequences) were subsampled 100 times from the data, and the average Chao1-estimate numbers of species were plotted for each increment. If a study sample had been thoroughly sequenced, the data plotted would approach an asymptotic plateau, indicating that further sequencing would not yield significantly more new species. This was done to confirm that the sequencing depth used in this study was adequate to capture the sample richness. [file 2049-2618-2-23-S2.pdf]

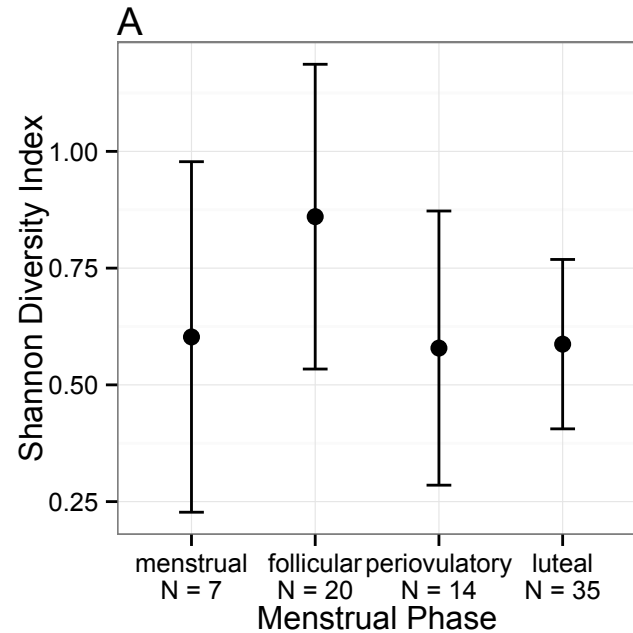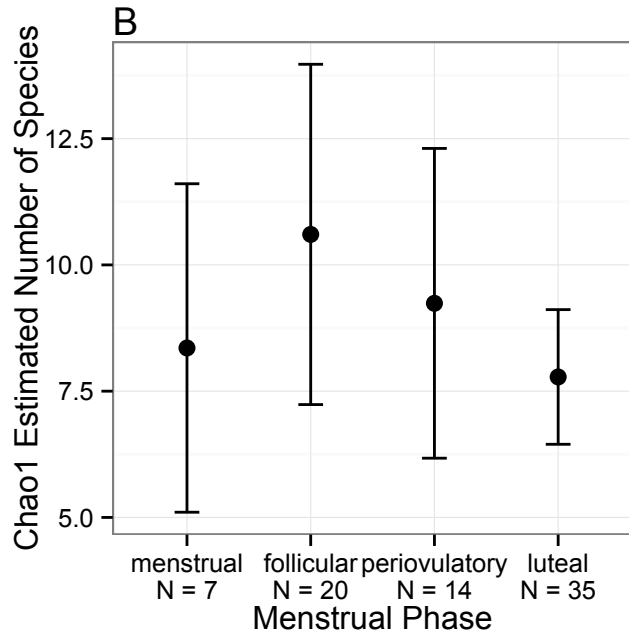

Supplement: Additional file 3 — Average Shannon Diversity and Chao1-estimated numbers of species by menstrual phase. Average Shannon Diversity (A) and Chao1-estimated number of species (B) by menstrual phase. Phases are defined as menstrual, day 1 (onset of menstruation) to cessation of bleeding (days 4 to 7); follicular, cessation of bleeding to day 12; periovulatory, day 13 to day 16; luteal, day 17 to day 26 to 32 (commencement of bleeding). The error bars indicate 95% confidence intervals. The only statistically significant difference determined was between the Chao1-estimated numbers of species between the follicular and luteal phases; however, we do not believe this to be biologically significant. [file 2049-2618-2-23-S3.pdf]

## A. Bootstrapped Shannon Diversity Index

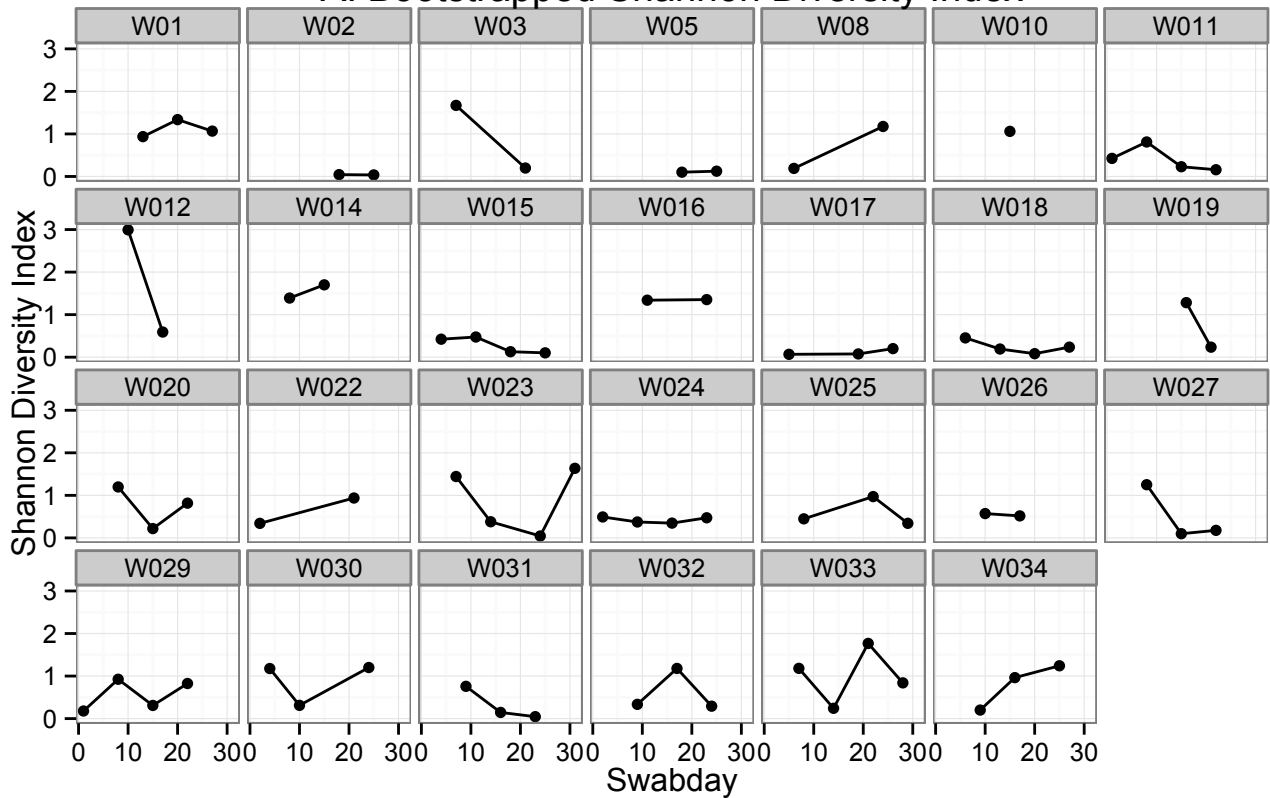

## B. Bootstrapped Chao1 Estimates

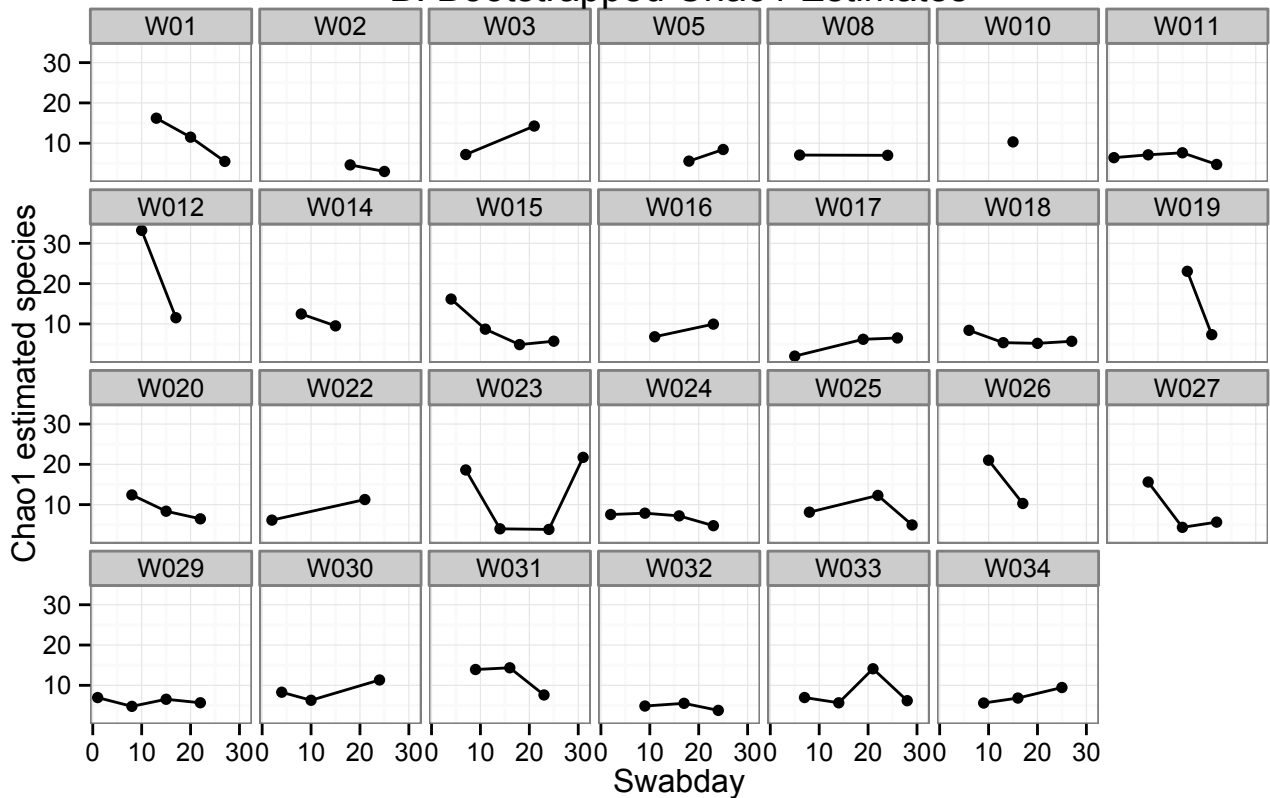

Supplement: Additional file 5 — Temporal variation in Bootstrapped Shannon diversity index and Chao1 estimates for each individual. Graphs showing the change in bootstrapped Shannon diversity index (A) and Chao1 estimates (B) for each woman (N = 27) by the day each sample was taken. The plots reflect the findings that many women had diversity statistics that remained consistent throughout the study period, whereas some women had changes in the values of these measures. [file 2049-2618-2-23-S5.pdf]
